# Enhancing genomic selection for reproductive traits in turkeys through SNP prioritization using the fixation index

**DOI:** 10.1016/j.psj.2025.106213

**Published:** 2025-12-08

**Authors:** Evan Hartono, Owen W. Willems, Xuechun Bai, Benjamin J. Wood, Sajjad Toghiani, Romdhane Rekaya, Samuel E. Aggrey

**Affiliations:** a Department of Poultry Science, University of Georgia, Athens, GA 30602, USA; b Hybrid Turkeys, Kitchener, Ontario, Canada; c School of Veterinary Science, The University of Queensland, Gatton Campus, QLD 4343, Australia; d USDA, Agricultural Research Service, Animal Genomics and Improvement Laboratory, Beltsville, MD 20705, USA; e Department of Animal and Dairy Science, University of Georgia, Athens, GA 30602, USA

**Keywords:** fixation index, genomic selection, turkey, reproductive traits

## Abstract

Genomic selection (GS) has revolutionized the field of animal breeding by enabling more accurate estimates of breeding values through a more precise assessment of the additive relationships between individuals. For lowly heritable traits, the increase in accuracy was in general modest at best. This is specifically the case for reproductive traits in turkeys which also suffer from a lack of normality of their phenotypic distributions. This study aimed to increase the accuracy of genomic selection for reproductive traits in turkeys by enhancing the quality of the genomic relationship matrix and by improving the normality of the phenotypic distributions. The fixation index (F_ST_) was used to prioritize and select informative subsets of single nucleotide polymorphisms (SNPs) associated with genetic differentiation between extreme phenotypic groups. Concurrently, the Box-Cox transformation was applied to improve the normality of the reproductive trait phenotypic distributions. A total of 6,103, 5,564, and 5,548 records for egg production percent (PEP), egg fertility (FERT) and fertile eggs hatchability (HOF) traits, respectively were analyzed using univariate pedigree-based BLUP (BLUP), genomic BLUP (GBLUP), and single-step GBLUP (ssGBLUP) approaches. Incorporating F_ST_-prioritized SNPs generally improved heritability estimates, with substantial gains observed for the lowly heritable fertility (FERT) trait when combined with the ssGBLUP approach. SNPs were prioritized using the 90th, 95th, and 99th percentiles of the F_ST_ distribution (F_ST_90, F_ST_95, and F_ST_99). These thresholds retained approximately 4,801 SNPs (10 %), 2,400 SNPs (5 %), and 480 SNPs (1 %), respectively, out of the 48,008 markers available after quality control. For FERT, using all SNPs in GBLUP produced a baseline heritability of 0.15, whereas prioritizing the top 10 % F_ST_ markers increased the estimate to 0.29 (+93 % gain) for untransformed data. The ssGBLUP model also showed improvement, with heritability increasing from 0.12 (baseline) to 0.18 **(+**50 % gain) under the F_ST_90 scenario. Furthermore, the Box-Cox transformation consistently resulted in higher heritability estimates across all methods compared to untransformed data, with ssGBLUP exhibiting the highest estimates after transformation. For FERT, the baseline GBLUP estimate increased from 0.15 (untransformed) to 0.19 after transformation **(**+27 % gain), while ssGBLUP increased from 0.12 to 0.15 **(**+25 % gain). When Box–Cox transformation was combined with F_ST_90, heritability further increased to 0.33 for GBLUP **(**+74 % relative to its transformed baseline**)** and 0.21 for ssGBLUP (**+**40 % relative to its transformed baseline**)**. The high re-ranking in the top 10 % after using SNP-prioritization suggests that marker filtering is likely to improve the accuracy of the selection decision through the correct identification of superior turkeys. These findings highlight the potential of F_ST_-based SNP prioritization and data transformation techniques to enhance the accuracy of genomic selection for reproductive traits in turkeys.

## Introduction

Traditional methods for the genetic improvement of farm animals have relied on trait phenotypes and pedigree information. Even with the most comprehensive pedigree data available, the accuracy of estimated breeding values (EBVs) is generally moderate to low, especially for animals with limited information and traits of low heritability. Genomic information allows for the estimation of realized relationships between individuals and has increased the accuracy of breeding values estimation and the correction of pedigree errors and unreported family relationships (Meuwissen and Goddard, 1996). The cost of genotyping has dropped since 1990 and is still decreasing rapidly with the continuous development of technology, which allows more animals to be genotyped to further increase accuracy. However, when the number of genotyped animals is fixed, only increasing the number of genetic markers beyond certain points does not improve the accuracy of estimating the observed genetic relationship matrix (Su et al., 2012). One way to further improve the accuracy of genomic selection is through the optimization of the genetic relationship matrix (**G**). This could be achieved by using a subset of prioritized SNP markers to construct **G** that strengthens the association between the phenotypic traits and the genomic data (Chang, 2018)).

Traditionally, SNP filtering is conducted based on certain statistical criteria such as p-values for single-marker analyses or quality of fit and model determination for Bayesian procedures such as BayesB (Meuwissen et al., 2001) and BayesR (Erbe et al., 2012). The performance of these methods is hampered, at different degrees, by the high false positive rate, multiple testing problems, high linkage disequilibrium (LD), and the small SNP effects. It is frequently the case that there is not enough statistical power to prioritize between hundreds of markers that are in LD with a QTL explaining a very small fraction of the genetic variance.

MacLeod et al. (2016) presented an extension of BayesR through the inclusion of biological prior information (variant type, location in differentially expressed genes) in the prioritization process but it was only marginally better than BayesR (Erbe et al., 2012).

The Fixation Index (F_ST_) measures the genetic differentiation between subpopulations relative to the total population. Developed by Sewall Wright, F_ST_ is derived from his F-statistics, which partition the genetic variation within these populations. A higher F_ST_ value for all three reproductive traits indicates greater genetic divergence among subpopulations, potentially due to reduced gene flow, adaptation to local environments, or the action of divergent selection pressures. F_ST_ has wide applications in population genetics, conservation biology, and evolutionary studies, as it provides insights into the genetic structure, migration patterns, and evolutionary history of populations (Wright, 1965; Weir and Cockerham, 1984). Lewontin and Krakauer (Lewontin and Krakauer, 1973) showed that differences in allele frequencies between subpopulations, quantified by the F_ST_ statistic, arise because of selective forces acting on these populations. Consequently, F_ST_ can be utilized to identify SNPs under selective pressure due to their LD with quantitative trait loci (QTL).

Toghiani et al. (2017) proposed an innovative approach that employs the F_ST_ statistic to filter high-density SNP data. Through simulated data, they showed that F_ST_ scores can successfully detect QTL, particularly those with moderate to large effects on traits. Utilizing only 2.5 % of the genotyped SNPs that were prioritized based on their F_ST_ scores enabled effective tagging of most QTL across various chromosomes, frequently with multiple SNPs associated with each QTL. The genomic similarity computed using the selected SNPs was high (greater than 0.80) for individuals sharing similar genetic and phenotypic characteristics, even in cases where they had a limited or non-existent pedigree relationship.

**Fig. 1:**
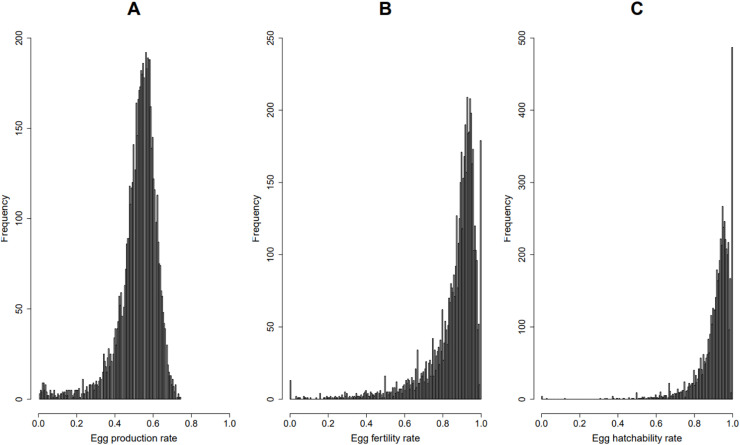
Distributions of raw data for egg production rate (A), egg fertility rate (B), and egg hatchability rate (C).

In turkeys, the phenotypic distributions of FERT and HOF show a significant departure from normality while PEP shows a slight departure as shown in Figure 1. Data transformation is a technique frequently employed to improve the distribution of trait phenotypes to conform with the assumed normality of the data used in genetic evaluation models. Research comparing raw and transformed phenotypic data in chickens using BLUP revealed significant change in heritability estimates and the ranking of individuals (Besbes et al., 1993).

The goal of this study is to investigate the possible improvements in the accuracy of genomic prediction through the improvement of the genomic relationship matrix (G) and the transformation of phenotypic data of non-normally distributed reproductive traits. The fixation index is used to prioritize a subset of SNP markers that are potentially in high LD with QTL. This prioritized set of SNPs will be used to compute G. Additionally the Box-Cox transformation is used to enhance the phenotypic distribution of the analyzed three reproductive traits. The Box-Cox transformation is a proven method used to improve the distribution of egg production traits and subsequently genetic parameters (Besbes et al., 1993; Ünver et al., 2002; Chen and Tixier-Boichard, 2003; Ünver et al., 2004; da Silva et al., 2012). Although the field of variant prioritization remains very active with the expanded availability of high-density marker data and sequence information, it is not our objective in this study to compare existing methods. The F_ST_ approach is at least as good as other existing methods. Similarly, there are many transformations that can be used to improve the normality of the analyzed traits.

## Materials and methods

### Phenotypic and genomic data

Egg production percent (PEP), egg fertility (FERT), and hatch of fertiles (HOF) collected between 2009 and 2018 were used. A data set of 836,784 animals (376,797 females with 1,461 missing records and 459,987 males with 1,614 missing records), was used for the pedigree analysis using Optisel package (Wellmann, 2019) in R 4.4.1 software (R Core Team, 2024). Pedigree depth and quality were assessed using equivalent complete generations (ECG), fully traced generations, maximum generations traced, pedigree completeness index (PCI), and inbreeding coefficients. On average, animals had 15.46 equivalent complete generations. The pedigree completeness index was high (mean = 0.93; median = 1.0), indicating that most pedigrees were nearly complete. Analysis of family structure revealed 3,754 sire families with an average of 222 offspring per sire (maximum 770, minimum 1), and 18,474 dam families with an average of 45 offspring per dam (maximum 125, minimum 1).

The phenotypic data consisted of 6,103, 5,564, and 5,548 records for PEP, FERT and HOF, respectively. To avoid redundancy, detailed phenotypic descriptive statistics for FERT, PEP, and HOF from the same population and dataset are provided in (Hartono et al., 2025), and the phenotypic values remain identical across the F_ST_ clusters because only the genotypic information was a subset. The phenotypic values for the three traits ranged between 0 and 1. The genomic data consisted of the genotypes of 61,705 SNP markers collected on 17,104 turkeys. Missing SNP genotypes were imputed using FImpute software (Sargolzaei et al., 2014). After quality control using the QCF90 software (Masuda et al., 2019), 13,956 turkeys and 48,008 SNPs were retained. Markers were excluded if they had call rate < 0.95, minor allele frequency (MAF) < 0.01, deviations from Hardy–Weinberg equilibrium (*p* < 1 × 10⁻⁶), or pedigree-inconsistent genotypes. From this set, the F_ST_90, F_ST_95, and F_ST_99 thresholds retained approximately 4,801**,** 2,400, and 480 SNPs, respectively. The number of genotyped turkeys with phenotypic records for PEP, FERT, and HOF was 2,075, 1,443, and 1,440, respectively. Descriptive statistics of the data are shown in Table 1.

**Table 1:** Summary statistics of the reproductive traits^1^ (%).

Trait^1^	N	Mean	SD	CV	Min	Max
PEP	6103	0.52	0.10	20.10	0	0.74176
FERT	5564	0.86	0.14	16.01	0	1
HOF	5548	0.91	0.09	9.61	0	1

1 PEP: Egg production percent, FERT: Egg fertility, HOF: Egg hatchability, SD: Standard Deviation, CV: Coefficient of Variation; Trait values are in proportion (0-1) scale.

### Statistical model and data analysis

The Box-Cox transformation is a statistical technique that uses power transformation to improve normality (Box and Cox, 1964). It is a flexible, data-driven approach to identify the optimal normalizing transformation from a family of power transformations.

The data for each trait (transformed and untransformed) were analyzed using BLUP, GBLUP, and ssGBLUP as implemented in BLUPF90 (Misztal et al., 2014). The following univariate mixed linear model was used:

$$y_{ij}=H_{i}+a_{j}+e_{ij}$$

where $y_{ij}$ is the observed phenotype for FERT, PEP, or HOF for bird *j, H_i_* was the fixed effect of the hatch year class *i*,$\;a_{j}$ is the random additive effect of bird *j*, and $e_{ij}\;$is the random residual term. The additive effects were assumed to be normally distributed:

$$a={\left(a_{1},a_{2},\;\ldots ,a_{n}\right)}^{\prime }∼N\left(0,V\right)$$

where $V={A\sigma }_{a}^{2}$ for BLUP, $V={G\sigma }_{a}^{2}$, for GBLUP and $V={H\sigma }_{a}^{2}$ for ssGBLUP with **A** and **G** being the average and observed additive relationship matrices, and **H** is a blended matrix of **A** and **G**, $\sigma_{a}^{2}$ is the genetic variance. All the analysis was carried out using BLUPF90 (Misztal et al., 2014).

### SNP prioritization based on F_ST_ scores

The genotyped turkeys were clustered into three sub-populations based on the phenotypic distribution of each trait. Subpopulation 1 (S1) included 5 % of the turkeys with the lowest phenotypes and subpopulation 2 (S2) consisted of 5 % of the turkeys with the highest phenotypes. The third subpopulation (S3) included the remaining turkeys. For each SNP marker, the F_ST_ score was calculated as:

$$F_{ST}\;=\;\frac{H_{T}\;-\;H_{s}}{H_{T}}$$

where $H_{T}$ = 2 * p * q is the expected heterozygosity in the population, and $H_{s}$ the average expected heterozygosity across the subpopulations and it is given by:

$$H_{s}\;=\;\frac{H_{S1}*\;n_{s1}\;+\;H_{S2}\;*\;n_{s2})}{\left(n_{s1}\;+\;n_{s2}\right)\;}$$

with$\;H_{Si}\;=\;2\;*\;p_{Si}\;*q_{Si}$ where $p_{Si}$ and $q_{Si}$ are the allele frequencies in subpopulation i and $n_{s1}$ and $n_{s2}\;$are the number of individuals in subpopulations 1 and 2, respectively. A low F_ST_ value indicates similar allele frequencies across the subpopulations at that locus. A high F_ST_ suggests a lack of shared alleles, which can be a signature of positive directional selection acting on that locus between the extreme phenotypic groups. The rank correlation as implemented in R (R Core Team, 2014) was used to assess the change in individuals ranking and possible breeding decisions due to SNP prioritization and phenotypic transformation. To assess re-ranking across scenarios, Spearman's rank correlation is computed between the untransformed BLUP breeding values and other scenarios, considering both the top 10 % of turkeys and the entire population.

## Results

Estimates of variance components and heritability for PEP, FERT, and HOF using the three methods (BLUP, GBLUP, and ssGBLUP) and the raw data (non-transformed phenotypes) are presented in Tables 2–4. Using GBLUP, heritability estimates were numerically smaller for PEP and HOF and larger for FERT compared to BLUP and ssGBLUP, although the differences are not statistically significant due to the large standard errors.

**Table 2:** Genetic parameter estimates of the egg production percent (PEP) using BLUP, GBLUP, and ssGBLUP.

	BLUP			GBLUP			ssGBLUP		
	$\sigma_{a}^{2}$	$\sigma_{e}^{2}$	h^2^ ± SE	$\sigma_{a}^{2}$	$\sigma_{e}^{2}$	h^2^± SE	$\sigma_{a}^{2}$	$\sigma_{e}^{2}$	h^2^ ± SE
Non transformed data									
Baseline^1^	0.0026	0.0084	0.23 ± 0.03	0.0020	0.0085	0.19 ± 0.04	0.0026	0.0084	0.24 ± 0.03
F_ST_90				0.0022	0.0084	0.21 ± 0.03	0.0018	0.0092	0.25 ± 0.03
F_ST_95				0.0020	0.0086	0.19 ± 0.03	0.0015	0.0094	0.23 ± 0.02
F_ST_99				0.0010	0.0095	0.10 ± 0.02	0.0017	0.0092	0.16 ± 0.02
Box-Cox transformed data									
Baseline^1^	0.0006	0.0015	0.30 ± 0.03	0.0005	0.0014	0.27 ± 0.04	0.0007	0.0014	0.31 ± 0.03
F_ST_90				0.0006	0.0012	0.33 ± 0.04	0.0008	0.0014	0.36 ± 0.03
F_ST_95				0.0004	0.0014	0.24 ± 0.03	0.0007	0.0014	0.32 ± 0.03
F_ST_99				0.0002	0.0016	0.13 ± 0.02	0.0006	0.0016	0.26 ± 0.03

1 Baseline: using all SNP_s_ for GBLUP and ssGBLUP_,_ F_ST_ : using prioritized SNP_s_ for F_ST_ scores higher than 10 %, 5 %, and 1 % quantiles. $\sigma_{a}^{2}$: genetic variance, $\sigma_{e}^{2}$: residual variance, h^2^ : heritability, SE: standard error.

Incorporating all available genomic marker data had varying impacts on the heritability estimates. For untransformed data, the baseline heritability estimates for the three reproductive traits were generally low to moderate. For FERT, heritability ranged from 0.11 to 0.19, with GBLUP providing a modest improvement over BLUP and ssGBLUP. PEP showed slightly higher baseline heritability estimates, ranging from 0.19 to 0.24, with ssGBLUP yielding the highest value. For HOF, baseline heritability ranged from 0.20 to 0.28, with BLUP and ssGBLUP producing similar estimates and GBLUP being slightly lower.

For untransformed FERT, including all markers in the GBLUP model led to a 36 % increase in heritability compared to the pedigree-based BLUP. In contrast, adding marker information resulted in a decrease heritability estimates for PEP (17 % lower) and HOF (29 % lower) relative to the BLUP model once again shown in Tables 2–4. When a subset of prioritized SNPs through the F_ST_ approach (F_ST_90, F_ST_95, and F_ST_99) was used to compute the genomic relationship matrix in GBLUP and ssGBLUP, variance components and heritabilities were altered (Tables 2–4). When 10 % of the SNPs were prioritized based on their F_ST_ scores (F_ST_90) the highest heritability estimates were observed across the three traits; with increases ranging between 3.6 % to 93 % compared to simply using all available genotypes. Similarly, using the top 5 % prioritized SNPs (F_ST_95) increased the heritability of FERT by 50 to 67 % and HOF by −14 to 25 %. However, prioritizing only 1 % of the SNPs (F_ST_99) resulted in a decrease in the heritability estimates for all three traits.

Tables 2–4 also present the estimates of variance components and heritabilities for PEP, FERT, and HOF using Box-Cox transformed phenotypes for the three different genetic evaluation approaches (BLUP, GBLUP, and ssGBLUP).

When the F_ST_90 were used to compute the G matrix after Box-Cox transformation, the heritability of FERT increased by 74 % and 40 % for GBLUP and ssGBLUP respectively when compared with using all available markers (baseline) (Table 2). Similar trends were observed for PEP and HOF with increased heritability estimates by 0 to 42 % shown in Table 3, Table 4, respectively. The F_ST_99 resulted in a decrease of heritability estimates except for FERT using ssGBLUP (Table 5).

**Table 3:** Genetic parameter estimates of the egg fertility (FERT) using BLUP, GBLUP, and ssGBLUP.

	BLUP			GBLUP			ssGBLUP		
	$\sigma_{a}^{2}$	$\sigma_{e}^{2}$	h^2^ ± SE	$\sigma_{a}^{2}$	$\sigma_{e}^{2}$	h^2^± SE	$\sigma_{a}^{2}$	$\sigma_{e}^{2}$	h^2^ ± SE
Non transformed data									
Baseline^1^	0.0019	0.0146	0.11 ± 0.02	0.0026	0.0145	0.15 ± 0.05	0.0022	0.0160	0.12 ± 0.03
F_ST_90				0.0048	0.0119	0.29 ± 0.04	0.0033	0.0150	0.18 ± 0.03
F_ST_95				0.0041	0.0123	0.25 ± 0.04	0.0034	0.0149	0.18 ± 0.03
F_ST_99				0.0022	0.0140	0.14 ± 0.03	0.0025	0.0157	0.14 ± 0.02
Box-Cox transformed data									
Baseline^1^	0.0011	0.0075	0.13 ± 0.02	0.0016	0.0068	0.19 ± 0.05	0.0013	0.0073	0.15 ± 0.03
F_ST_90				0.0028	0.0056	0.33 ± 0.04	0.0018	0.0069	0.21 ± 0.03
F_ST_95				0.0024	0.0060	0.29 ± 0.04	0.0019	0.0069	0.21 ± 0.03
F_ST_99				0.0014	0.0070	0.16 ± 0.03	0.0015	0.0072	0.17 ± 0.03

1 Baseline: using all SNP_s_ for GBLUP and ssGBLUP, F_ST_: using prioritized SNP_s_ for F_ST_ scores higher than 10 % (F_ST_90), 5 % (F_ST_95), and 1 % (F_ST_99) quantiles. $\sigma_{a}^{2}$: genetic variance, $\sigma_{e}^{2}$: residual variance, h^2^: heritability, SE: standard error.

**Table 4:** Genetic parameter estimates of the egg hatchability (HOF) using BLUP, GBLUP, and ssGBLUP.

	BLUP			GBLUP			ssGBLUP		
	$\sigma_{a}^{2}$	$\sigma_{e}^{2}$	h^2^ ± SE	$\sigma_{a}^{2}$	$\sigma_{e}^{2}$	h^2^± SE	$\sigma_{a}^{2}$	$\sigma_{e}^{2}$	h^2^ ± SE
Non transformed data									
Baseline^1^	0.0022	0.0056	0.28 ± 0.03	0.0019	0.0072	0.20 ± 0.05	0.0022	0.0056	0.28 ± 0.03
F_ST_90				0.0027	0.0064	0.30 ± 0.05	0.0022	0.0055	0.29 ± 0.03
F_ST_95				0.0022	0.0068	0.25 ± 0.04	0.0019	0.0058	0.24 ± 0.03
F_ST_99				0.0011	0.0078	0.13 ± 0.03	0.0010	0.0065	0.14 ± 0.02
Box-Cox transformed data									
Baseline^1^	0.0014	0.0034	0.29 ± 0.03	0.0013	0.0040	0.24 ± 0.05	0.0015	0.0033	0.31 ± 0.03
F_ST_90				0.0018	0.0035	0.34 ± 0.05	0.0015	0.0033	0.31 ± 0.03
F_ST_95				0.0014	0.0038	0.26 ± 0.04	0.0014	0.0035	0.28 ± 0.03
F_ST_99				0.0007	0.0044	0.14 ± 0.03	0.0008	0.0039	0.17 ± 0.02

1 Baseline: using all SNP_s_ for GBLUP and ssGBLUP_,_ F_ST_: using prioritized SNP_s_ for F_ST_ scores higher than 10 % (F_ST_90), 5 % (F_ST_95), and 1 % (F_ST_99) quantiles. $\sigma_{a}^{2}$: genetic variance, $\sigma_{e}^{2}$: residual variance, h^2^: heritability, SE: standard error.

**Table 5:** Rank correlations for the top 10 % and all animal on PEP, FERT, and HOF across six different scenarios.

		Rank Correlations					
	Top 10 % of population					100 % of population	
Trait^7^	r_12_	r_13_	r_14_	r_15_	r_16_	r_15_	r_16_
PEP	0.987	0.999	0.856	0.645	0.541	0.921	0.935
FERT	0.977	0.989	0.832	0.285	0.311	0.940	0.923
HOF	0.995	0.999	0.915	0.404	0.438	0.942	0.945

1: Untransformed data with BLUP, 2: Transformed data with BLUP, 3: Untransformed data with ssGBLUP, 4: Transformed data with ssGBLUP, 5: Untransformed data with ssGBLUP and FST90, 6: Transformed data with ssGBLUP and FST90.

7 FERT: Percent of fertile eggs, PEP: Percent egg production, HOF: Hatch of fertile.

Table 5 presents the rank correlations of breeding values for all and top 10 % turkeys and for each of the three reproductive traits (PEP, FERT, and HOF) using all or 10 % of the prioritized SNPs (F_ST_90), regular BLUP or ssGBLUP, and transformed or non-transformed phenotypes. For F_ST_90, the correlations between breeding values obtained using BLUP with or without the phenotype transformation (r12 in Table 5) ranged between 0.98 and 1. The largest re-ranking was observed for FERT (*r* = 0.977). The rank correlation between breeding values using BLUP and ssGBLUP varied between 0.99 and 1 for non-transformed phenotypes (r13). Using non-transformed data for BLUP and transformed data from ssGBLUP, the correlation was between 0.83 and 0.92 (r14 in Table 5). When only 10 % of the SNPs were prioritized (F_ST_90), the correlation between breeding values obtained using BLUP with non-transformed phenotypes and ssGBLUP (non-transformed data) ranged between 0.29 and 0.65 (r15 in Table 5) and 0.31 to 0.54 using BLUP with non-transformed and ssGBLUP with transformed records (r16 in Table 5), respectively. In both cases, the highest correlation was for PEP. These results indicated a substantial re-ranking of top individuals largely due to the preselection of SNP markers to compute the G matrix.

## Discussion

There are relatively few published estimates available for the genetic parameters of these reproductive traits in turkey populations. The heritability estimates obtained for PEP, FERT, and HOF in this study were generally similar to values previously reported in the literature (Case et al., 2010; Proulx et al., 2014; Makanjuola et al., 2022) with some variations due to the use of transformation, different recording periods of the records, and different models being used in the analysis.

The impact of incorporating genomic marker data on heritability estimates varied across the traits. For FERT, the use of genomic information through GBLUP substantially improved the heritability estimate, suggesting that markers captured additional genetic variance not accounted for by pedigree relationships alone.

F_ST_ was calculated using equal-sized phenotypic tails (S1 and S2; each representing 5 % of the population) with allele frequencies estimated from a large sample size, conditions under which simple F_ST_ estimators are known to perform robustly. Because the two groups were balanced and no hierarchical population structure was present, bias due to unequal sample weighting which is one of the main advantages of the (Weir and Cockerham, 1984) estimator was minimized. Therefore, the estimator applied herein provides reliable and interpretable measures of between-group divergence for SNP prioritization.

The F_ST_-based approach to prioritize and select informative SNPs had mixed effects on heritability estimates. While the thresholds at the 90th and 95th percentiles of F_ST_ distribution generally improved heritability estimates, particularly for FERT compared to using all available markers included in both GBLUP and ssGBLUP, the more stringent 99th percentile threshold resulted in a decrease in heritability estimates for all traits. This is likely due to the inability of the very small subset of prioritized SNPs to tag the majority of the QTLs. Finding an optimal balance when prioritizing SNPs based on F_ST_ is crucial. Identifying informative markers can be challenging. Overly stringent thresholds risk the exclusion of potentially valuable markers, while loose thresholds allow for uninformative markers, weakening the signal from causative variants.

The ssGBLUP approach, which combines pedigree and genomic information, provided modest improvements in HOF and PEP heritabilities compared to pedigree-based BLUP. However, the substantial boost in FERT heritability achieved by incorporating F_ST_-prioritized SNPs does not increase the underlying additive genetic variance; instead, it reduces the downward bias inherent in genomic relationship matrix constructed from SNPs with very low minor allele frequencies or minimal between-individual contrast. When many such SNPs are included, the genomic relationships shrink toward zero. By prioritizing SNPs with higher F_ST_ values; those exhibiting meaningful allele frequency divergence between extreme phenotypic groups allows the genomic relationship matrix better reflects realized genetic relatedness. This results in a more accurate partitioning of variance between additive and residual components rather than an inflation of additive variance; allowing the genomic relationship matrix better capture the covariance structure already present in the population. In the F_ST_90 and F_ST_95 thresholds, most of the SNPs removed were those with minimal divergence and limited informativeness for estimating additive relationships. Filtering out these low-information markers reduces noise in the genomic relationship matrix and can increase the proportion of variance attributed to additive genetic effects as mentioned previously. In contrast, the F_ST_99 set retains only the 1 % most divergent SNPs, which typically exhibit allele frequencies approaching fixation. Near-fixed SNPs contribute little to within-population genetic variance, yielding a sparse genomic relationship matrix; reduced genomic contrast among individuals, additive variance estimates decreased. This explains the observed decline in heritability when using the F_ST_99 subset. Overall, the results demonstrate the potential of the F_ST_-based SNP prioritization method to enhance genomic selection accuracy, particularly for traits like FERT, where traditional GBLUP and ssGBLUP may not fully capture the genetic variance. The Box-Cox transformation improved the normality of the data and resulted in higher heritability estimates. The ssGBLUP method, which incorporates both pedigree and genomic data, performed best after transformation, suggesting that Box-Cox transformation could further improve the estimates by minimizing the non-normal distribution of the trait on the phenotypic level. The Box–Cox transformation improves the statistical properties of the phenotype so the linear mixed model can more accurately partition variance components. Many reproductive traits, including the ones analyzed herein, exhibit pronounced skewness and heteroscedasticity. Under these conditions, the residuals deviate from normality and the model tends to inflate the residual variance component to absorb distributional irregularities. By stabilizing the variance and reducing skewness, the Box–Cox transformation produces residuals that better satisfy the assumptions of the linear model. These results provide more precise estimation of the residual variance and reduce the amount of true additive variation that is inadvertently assigned to the residual term. Consequently, the proportion of variance attributed to the additive genetic effect increases, yielding more reliable heritability estimates. The effectiveness of the Box–Cox transformation for reproductive and fitness traits in turkeys, including the associated improvements in distributional shape for the same dataset being used in the current study has been demonstrated in detail in (Hartono et al., 2025). Box-Cox transformation and the choice of BLUP or ssGBLUP model had minimal impact on the ranking of turkeys (Table 5). Although it has a limited effect on the ranking of all turkeys in the pedigree, the use of F_ST_-prioritized SNPs has led to more substantial re-ranking, for the top 10 % of the turkeys particularly for the lowly heritable FERT trait (Table 5). The high degree reranking from incorporating F_ST_90 for both transformed and untransformed in the top 10 % means selecting the wrong individuals and their offspring are more likely to be selected as future breeding stock. The misidentified superior individuals can perpetuate suboptimal genetic trends, hampering the rate of genetic improvement over multiple generations. While the re-ranking effects were less pronounced for the more heritable traits, such as PEP and HOF, the inclusion of F_ST_-prioritized SNPs still resulted in some re-ranking within the top tier. This finding indicates that even for lowly to highly heritable traits, there is potential for improving selection accuracy and genetic gains through SNP prioritization and data transformation.

Filtering SNPs based on high F_ST_ values also has implications for inbreeding and long-term genetic variance. Because F_ST_ prioritizes loci with strong allele frequency divergence between the most extreme phenotypes, the retained markers tend to exhibit more polarized allele frequencies than the genome as a whole. When the genomic relationship matrix is constructed from such SNPs, individuals may appear more differentiated than they truly are on a genome-wide basis, potentially inflating estimated inbreeding coefficients or exaggerating differences among families. Conversely, if too few SNPs are retained (e.g., F_ST_99), the genomic relationship matrix may fail to capture background relatedness, resulting in unstable or biased inbreeding estimates. Over multiple generations, strong emphasis on high F_ST_ loci could accelerate fixation in already divergent genomic regions, reducing available additive genetic variance. Thus, while prioritization improves signal-to-noise ratio and reduces downward bias in variance estimation, breeding programs must balance SNP prioritization with maintaining genome-wide coverage to avoid distorting inbreeding estimates or compromising long-term genetic variation.

## Conclusion

This study investigated the use of the fixation index to prioritize informative SNPs and the Box-Cox transformation to improve the normality of the phenotypic distributions in estimating genetic parameters for reproductive traits in turkeys. The F_ST_-based SNP prioritization effectively captures subsets of markers associated with genetic differentiation between extreme phenotypic groups. Incorporating these prioritized SNPs into genomic prediction models generally improved heritability estimates, particularly for the lowly heritable FERT trait. The substantial boost in FERT heritability achieved by combining F_ST_-prioritized SNPs with the ssGBLUP approach highlights the potential of this method to capture additional genetic variance.

Furthermore, the Box-Cox transformation improved the normality of the reproductive trait data distributions. The transformed data yielded consistently higher heritability estimates across all methods (BLUP, GBLUP, and ssGBLUP) compared to the untransformed data. The ssGBLUP approach exhibited the highest heritability estimates after Box-Cox transformation, highlighting the synergistic benefits of combining data transformation with SNP prioritization method. By optimizing the genomic relationship matrix and improving the distributional properties of the phenotypes, the combined use of F_ST_-based SNP prioritization and Box–Cox transformation can enhance the precision of breeding value estimation in commercial turkey breeding programs. However, the balance of SNP retention is critical: moderate F_ST_ thresholds (e.g., F_ST_90–95) mainly remove low-information, weakly differentiated markers, which can reduce noise and strengthen the genomic signal. In contrast, extremely stringent thresholds such as F_ST_99 retain mainly near-fixed SNPs, which contribute little to within-population additive variance and can therefore reduce heritability estimates. These dynamics underscore the importance of selecting F_ST_ thresholds that improve signal quality without discarding markers relevant to the polygenic architecture of reproductive traits. The substantial re-ranking observed in this study illustrates that integrating F_ST_-guided SNP selection with appropriate trait transformation can alter breeding value predictions substantially, particularly among top candidates targeted for selection. The study provides a novel and actionable framework for optimizing genomic evaluation pipelines in commercial turkey breeding programs.

## Disclosures

The authors declare no competing interests

## Ethics approval and consent to participate

The data forms part of routine data collection by Hendrix Genetics. Thus, an IACUC protocol is not applicable.

## Consent for publication

All authors have read and agreed to publish the research reported in this work

## Funding

No funding was received for this work

## Availability of data and materials

The data that support the findings of this study are available from Hybrid Turkeys upon reasonable request, but restrictions apply to their availability, because they were used under a license of a material transfer agreement for the current study, and thus are not publicly available.

## CRediT authorship contribution statement

**Evan Hartono:** Writing – review & editing, Writing – original draft, Investigation, Formal analysis. **Owen W. Willems:** Writing – review & editing, Resources, Data curation. **Xuechun Bai:** Writing – review & editing, Resources, Data curation. **Benjamin J. Wood:** Writing – review & editing, Resources, Data curation. **Sajjad Toghiani:** Writing – review & editing, Investigation, Formal analysis. **Romdhane Rekaya:** Writing – review & editing, Supervision, Methodology, Investigation. **Samuel E. Aggrey:** Writing – review & editing, Supervision, Resources, Project administration, Investigation, Conceptualization.
